# mHealth Monitoring of Treatment of Cutaneous Leishmaniasis Patients: A Community-Based Implementation Study

**DOI:** 10.4269/ajtmh.22-0805

**Published:** 2023-08-28

**Authors:** Alexandra Cossio, Martha Milena Bautista-Gomez, Neal Alexander, Alejandra María del Castillo, María del Mar Castro, Patricia Yaneth Castaño-Grajales, Yeison Hawer Gutiérrez-Poloche, Laura Sofía Zuluaga, Leonardo Vargas-Bernal, Andrés Navarro, Nancy Gore Saravia

**Affiliations:** ^1^ Centro Internacional de Entrenamiento e Investigaciones Médicas, Cali, Colombia;; ^2^ Universidad Icesi, Cali, Colombia;; ^3^ Universidad Icesi, Grupo i2t, Cali, Colombia

## Abstract

Cutaneous leishmaniasis (CL) remains a global health problem. Compelled by the protracted healing process, initial and final outcomes of treatment are determined at 90 and 180 days, respectively, after initiation of treatment. Loss to follow-up during these intervals is substantial. Consequently, the effectiveness of treatment is largely unknown. We conducted an effectiveness-implementation hybrid design study of a community-based mobile health (mHealth) strategy to monitor adherence to anti-leishmanial treatment, adverse drug reactions, and therapeutic response compared with standard of care in two rural communities of Colombia. Three implementation outcomes were evaluated: usability and acceptability by qualitative methods and fidelity using quantitative methods. Fifty-seven patients were prospectively included in the mHealth intervention and 48 in the standard-of-care group. In addition, 24 community health leaders (CHLs), health workers, and patients participated in qualitative evaluations. The intervention significantly increased the proportion of patients having follow-up of therapeutic outcomes 90 and 180 days after initiating treatment from 4.2% (standard of care) to 82.5% (intervention), *P* < 0.001. The proportion of patients having records of treatment adherence, adverse drug reactions, and therapeutic response also increased significantly (*P* < 0.001). Fidelity to the intervention (recording of treatment adherence, adverse drug reactions, lesion photographs, and evaluation of therapeutic response) was 70–100%. The app was highly accepted by CHLs, health workers, and patients, who perceived that the app improved case identification and follow-up and met a public health need. Although usability was high, low connectivity affected real-time transmission of data. This community-based mHealth strategy facilitated access to health care for CL in rural areas and knowledge of treatment effectiveness.

## INTRODUCTION

Neglected tropical diseases (NTDs) affect more than one billion people worldwide who live in conditions of poverty. Among these illnesses, those that present with skin lesions, including cutaneous leishmaniasis (CL), are classified as skin NTDs.^1^^–^^3^ After Brazil, Colombia reports the second-highest number of cases of CL in the Americas (6,093 cases in 2021, incidence 22.7 per 100,000 inhabitants).^4^ Around 82% of patients with CL live in remote rural areas with limited access to a health system because of geographic, economic, and cultural barriers.^5^ Departments (first-level administrative divisions) with the highest incidence of CL in Colombia are Guaviare, Caldas, Santander, Tolima, and Risaralda, reporting annual incidences from 67 to 242 per 100,000 inhabitants.^5^

Standard of care for CL recommended by national guidelines includes weekly monitoring of treatment and evaluation of clinical response at 3, 6, and 12 months after treatment conclusion.^6^ Consequently, administration of treatment and follow-up of CL patients in remote rural areas is particularly challenging owing to the aforementioned barriers to access to health services.^7^ Currently, data on therapeutic responses to anti-leishmanial treatments in the Americas are largely limited to randomized clinical trials of efficacy; hence, the effectiveness of the diverse treatments administered is unknown.^4^

The use of mobile technologies has transformed some health services. Mobile health is defined as health practice supported by mobile devices such as mobile phones, patient monitoring devices, and personnel digital technologies.^8^ Mobile health (mHealth) has improved access to service, outcomes, and monitoring of visceral leishmaniasis in the community health system.^9^ Health interventions coupled with mHealth and community health worker participation have helped to bridge the gap between health providers and patients in dispersed rural areas.^3^^,^^9^^,^^10^

Mobile applications have also been developed to facilitate the diagnosis, clinical management, and epidemiological surveillance of CL. These apps are intended mainly for use by health professionals.^11^^–^^13^ The “Guaral” mHealth app for presumptive diagnosis of CL developed by our research team for use by community health volunteers was previously shown to reduce the time to diagnosis from 8 to 4 weeks.^14^^,^^15^ Recently, a complementary mobile app named “Guaral: seguimiento a tratamiento (Guaral+ST)” was designed and developed to facilitate monitoring of treatment and assessment of therapeutic response in CL patients. The high accuracy of the remote evaluation of therapeutic response using the photographs taken with the app was previously demonstrated.^16^

In the current study, an effectiveness-implementation hybrid design^17^^,^^18^ was used to assess the effectiveness of Guaral+ST for monitoring adherence to treatment, adverse reactions, and therapeutic response of patients with CL. Key implementation outcomes (fidelity, usability, and acceptability) are reported for patients in rural areas of two municipalities of Colombia having a high incidence of CL.

## MATERIALS AND METHODS

### Ethical approval and consent to participate.

This research was approved and monitored by the Institutional Ethical Review Board of the Centro Internacional de Entrenamiento e Investigaciones Médicas (CIDEIM) in accordance with national and international regulations (reference number 1295). Written informed consent was obtained from all participants, community health leaders (CHLs), health workers, and patients, as well as guardians of participants under 18 years of age. Assent was obtained from children over 7 years of age. Approval of the indigenous governor of the Emberá Chamí community in the Pueblo Rico study site was obtained prior to the initiation of the study, in addition to individual consent by community members. Waiver of informed consent was obtained from the institutional ethics committee for use of records of historical standard-of-care controls. This trial was registered with ClinicalTrials.gov (registration number NCT05533736).

### Study design.

**Figure 1.**
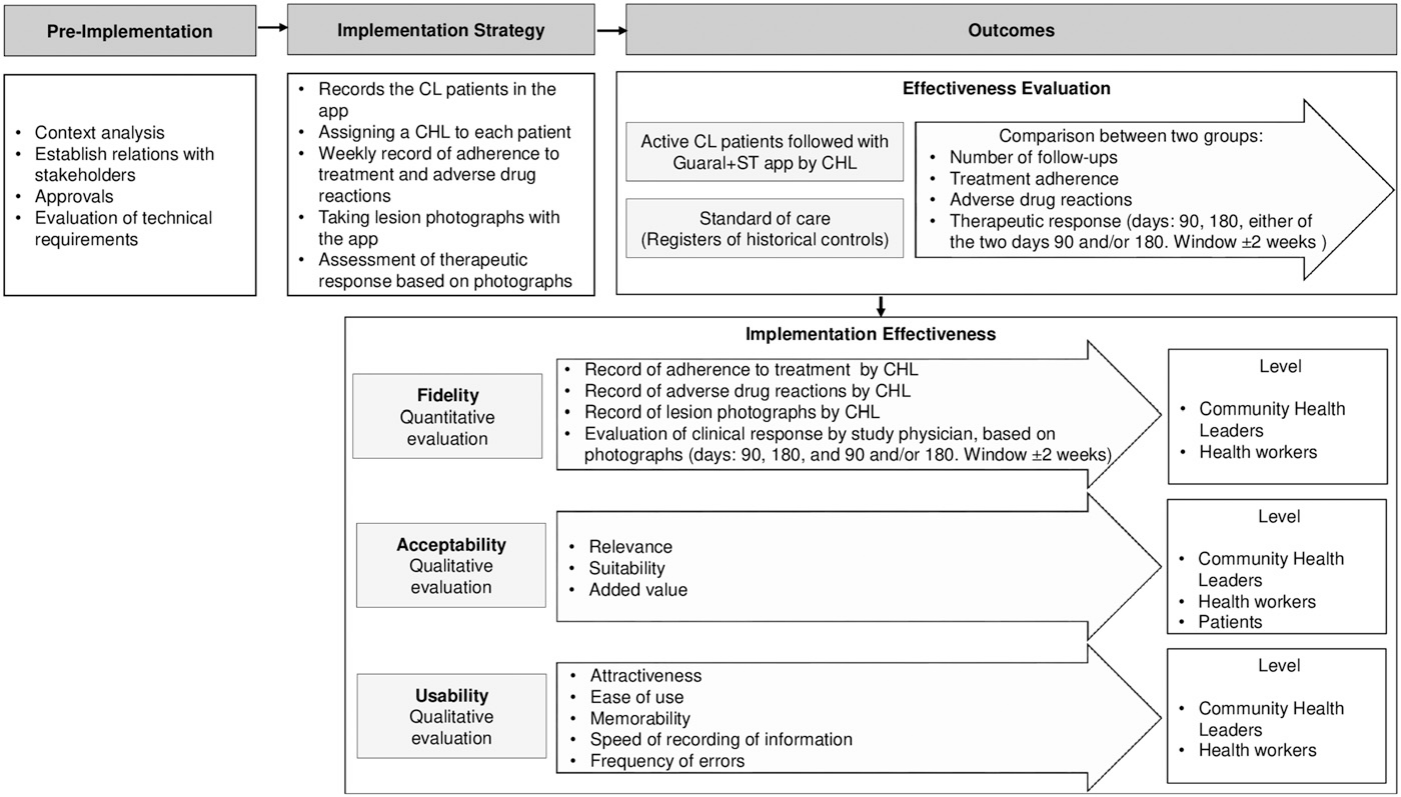
Study design. CHL = community health leader; CL = cutaneous leishmaniasis; Guaral+ST = Guaral: seguimiento a tratamiento.

An effectiveness-implementation hybrid type 2 design^18^^–^^20^ with a sequential explanatory mixed-methods approach was carried out between 2020 and 2021 at two study sites: Pueblo Rico, Risaralda and Rovira, Tolima. A quasi-experimental study with historical control (standard of care) was designed to evaluate the effectiveness of the mHealth intervention to improve monitoring of treatment of patients with CL by CHLs using the Guaral+ST app. The effectiveness indicators, including 1) number of follow-up contacts, 2) adherence to treatment, 3) adverse drug reactions, and 4) therapeutic response, were compared between the intervention and control groups. Acceptability and usability were evaluated qualitatively at three levels: CHLs, health workers, and patients. Fidelity was evaluated through quantitative methods (Figure 1). The study is reported in accordance with the Standards for Reporting Implementation Studies (StaRi) guidelines.^21^

### Study sites and context.

The study was conducted in two municipalities: Pueblo Rico in the Department of Risaralda and Rovira in the Department of Tolima. During 2020, Risaralda and Rovira reported CL incidences of 67.5 and 114 per 100,000 inhabitants, respectively; both were above the national average of 52.4 per 100,000 inhabitants.^5^

The town of Pueblo Rico is located on the eastern side of the western range of the Andes, (latitude 5.22156, longitude −76.0292), with a mean altitude of 1,560 meters above sea level (m.a.s.l.).^22^ This municipality extends 632 km^2^, and 99.9% of its territory is rural. Pueblo Rico is composed of 84 villages. Inhabitants belong to three main ethnic groups: indigenous (Emberá Chamí) (40%), Afro-Colombian (12%), and mestizo (48%).^23^ This diversity presents barriers for health interventions related to cultural, lingual, and literacy factors. Emberá is the indigenous language spoken; most of the indigenous population lives in remote mountain locations, and their access to and level of formal education are low.^7^ In contrast, the other two ethnic groups speak Spanish and customarily live closer to road infrastructure. The main health facility in Pueblo Rico, San Rafael Hospital, is located in the urban center. Participant recruitment for the study was conducted at the Santa Cecilia health post, which is the nearest health facility for most CL patients in the municipality. Personnel of the Santa Cecilia health post performed microscopic analysis of the direct smear of lesions to diagnose CL and medical evaluations prior to prescription of treatment.

The town of Rovira is located in the Department of Tolima, on the eastern slope of the central Andean range (latitude 4.23933, longitude −75.23968), having a mean elevation of 1,109 m.a.s.l.^22^ This municipality extends 736 km^2^ with a rural area comprising 99.9%.^24^ Rovira is composed of 82 villages; approximately 96.4% of the population is mestizo.^25^ San Vicente Hospital, the main health facility in the urban center, provides emergency and outpatient medical services as well as laboratory diagnosis, including microscopy of direct smear of lesions and pretreatment laboratory tests.

### Study participants.

Patients with active CL and historical records of patients with confirmed parasitological diagnosis were included in the quantitative evaluation. Further, a subgroup of CHLs, health workers, and patients participated in the qualitative evaluation. Eligibility criteria for study participants are summarized in Table 1.

**Table 1:** Inclusion criteria for participants in quantitative and qualitative evaluations

Inclusion criteria for participants in quantitative evaluation (effectiveness)	
Patients with active CL	Diagnosis confirmed by microscopy of tissue smear or culture
	Any age and gender
	Medical prescription for anti-leishmanial treatment
	Approved and signed informed consent
	Availability of CHL to monitor treatment of the patients in the community
Historical controls (standard of care)	Clinical records of patients with confirmed CL at most 2 years prior to the start of this study
	Any age and gender
	Received anti-leishmanial treatment based on national guidelines^6^
Inclusion criteria for participants in qualitative evaluations (acceptability and usability)	
CHLs	Residents of the rural areas of study sites
	Voluntary participation in the monitoring of patients using the mobile app
	Completion of the project training program on clinical aspects of CL, ethics, and use of the mobile app
	Had followed at least two patients using the app
	Signed and approved informed consent
Health workers	Professionals involved in the clinical management and assessment of the CL patients using the mobile app
	Approved and signed informed consent
Patients	Patients with active CL who were monitored by CHL using the app
	Approved and signed informed consent

CHL = community health leader; CL = cutaneous leishmaniasis.

### Sample size.

Sample size for the effectiveness evaluation was calculated using the Fleiss equation for differences between two proportions.^26^^,^^27^ The parameters were 95% confidence, 95% statistical power, and the expected proportions of patients with at least one follow-up estimated at 7% in the historical control group and 50% in the intervention (app) group.^28^ A ratio of two historical controls to one active CL case (2:1) was assumed, as were losses to follow-up of 20%. This yielded 48 historical controls and 24 patients with active CL. Active CL patients to be monitored using the app were enrolled consecutively, and historical records were selected by simple random sampling. For qualitative evaluation, the sample size of the active CL group was increased (*n* = 57) to include at least five CHLs at each study site who completed follow-up monitoring of at least two CL patients each. In addition, seven health workers who monitored treatment of patients with the app and six patients were included in the qualitative analysis.

### Intervention and implementation strategy.

Based on efficacy results of a previous study using the Guaral app for presumptive diagnosis and the Guaral+ST app by CHLs,^14^^–^^16^ expert consensus,^29^^,^^30^ and recommendations of the national guidelines,^6^ a community-based strategy utilizing the Guaral+ST app was undertaken to monitor the treatment and follow the therapeutic response of CL patients.

A context analysis was performed before beginning the study to appropriately build the relationship with health authorities, adapt the implementation strategy to the context, and identify and train CHLs. In addition, adjustments were made to the Guaral+ST mobile app to improve its operation in areas without Internet connectivity and to upload data and photos to the iCloud when and where access to connectivity by the users in the study sites was available.

**Figure 2.**
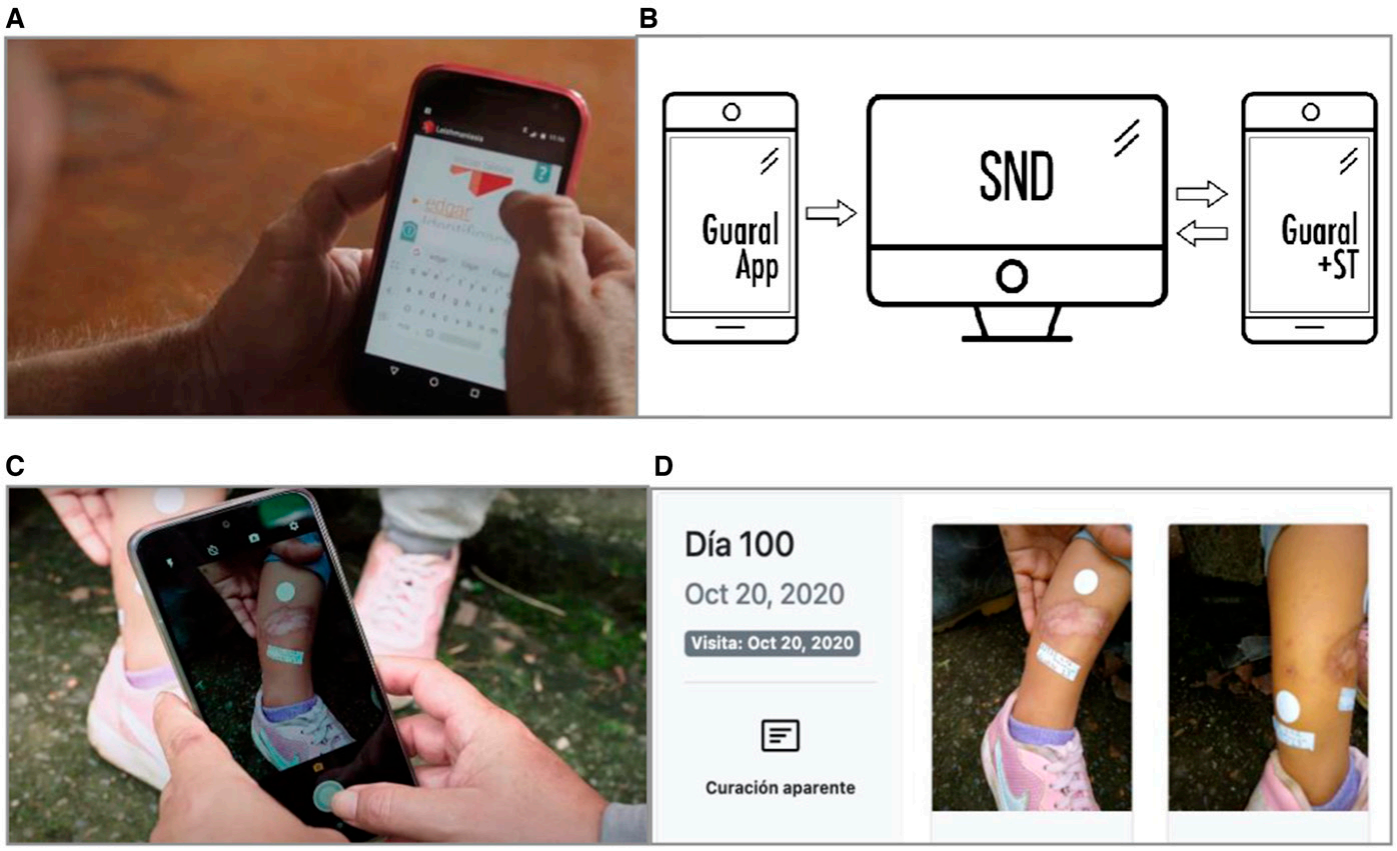
Schematic of implementation strategy. (**A**) Sociodemographic and clinical data and pretreatment lesion photographs of CL patients were recorded and transmitted to the cloud by the CHL using the Guaral app. (**B**) A CHL was assigned to each patient through the desktop Guaral: seguimiento a tratamiento (Guaral+ST) app by the field study coordinator using the System for Neurological Diseases (SND) app. (**C**) Weekly evaluation of adherence and adverse drug reactions was performed by the CHL in the Guaral+ST app. (**D**) Clinical response was evaluated by the study physician based on photographs taken by the CHL at the end of treatment and on days 90 and 180 ±2 weeks after initiating treatment using the Guaral+ST app. CHL = community health leader; CL = cutaneous leishmaniasis.

After confirmation of CL diagnosis by microscopy of tissue smear or culture, a prescription for treatment was issued by local hospital physicians in accordance with national guidelines.^6^ Active CL patients who met the inclusion criteria and provided informed consent were enrolled in the study. The strategy included the following steps. First, active CL patients were registered in the (Guaral) app for presumptive diagnosis by CHLs.^15^ Sociodemographic data as well as the number and localization of lesions were recorded, and photographs of lesions were taken before treatment was initiated. Second, a CHL was assigned by the field study coordinator to each patient based on place of residence through the System for Neurological Diseases (SND) desktop app.^15^ Third, during treatment, adherence and adverse drug reactions were recorded weekly by CHLs using the app. Photographs of lesions were taken at the end of treatment and on days 90 and 180 after initiation of treatment. Data and photographs were transmitted via the Internet to a cloud-based system. Finally, based on these photographs, the study physician evaluated the clinical response for each patient using the SND app (Figure 2).

To support the implementation of the strategy, CHLs and health workers were trained in ethical research conduct, clinical aspects of CL, and the study protocol and procedures and were evaluated on their knowledge and use of the Guaral+ST through accompaniment by the study coordinator when monitoring treatment with their first patients.

### Effectiveness of the community-based strategy with the Guaral+ST app for follow-up of CL patients.

Effectiveness was estimated by comparing the outcomes between active CL patients in the intervention group and those who previously received standard of care. The following proportions were compared between the two groups: 1) follow-up at end of treatment, day 90, day 180, and days 90 and/or 180 in accordance with the national guidelines for CL^6^; 2) number of follow-up visits after treatment; 3) record of adherence to treatment; 4) patients with adverse drug reactions recorded; and 5) record of therapeutic response at days 90 and/or 180. We considered windows of ±1 week for end of treatment and ±2 weeks for the last two visits to be acceptable.

Patient treatment was prescribed by the attending physician in the health facilities of the study sites in accordance with national guidelines.^6^ Systemic treatments administered during the study were meglumine antimoniate (MA), pentamidine, and miltefosine.

Initial and final cures were defined as the absence of new leishmaniasis lesions and complete re-epithelialization of the ulcers (based on photographs) at days 90 and 180 after initiating treatment, respectively. Therapeutic failure was defined as new lesions or less than 100% re-epithelization of lesions 90 or 180 days after initiating treatment.^29^ Patients who failed treatment were referred by the CHL to the local health facility for rescue treatment. Decisions on rescue treatment were made by the physicians at local health facilities on the basis of Colombian guidelines.^6^

### Implementation effectiveness.

Implementation outcomes were defined according to the Proctor model.^31^

#### Fidelity.

Defined as compliance with the implementation strategy as described in the protocol,^31^ fidelity was measured at two levels: CHL and physician. Fidelity was measured in terms of the following: 1) record of treatment adherence; 2) record of adverse drug reactions; 3) existence of photographs of lesions at day 90 or 180 ± 2 weeks; and 4) evaluation of therapeutic response. The CHLs were responsible for aspects 1 to 3, whereas aspect 4 was the responsibility of study physicians. Each area of fidelity was measured as a binary variable (either acceptable or unacceptable).

Treatment adherence and adverse drug reactions were evaluated weekly in the field while the patient received medication. The following numbers of evaluations were considered acceptable: three for MA, four for miltefosine, and two for pentamidine. Whether CHLs had obtained and recorded photographs was evaluated before treatment, at the end of treatment, and on or around days 90 and 180 after initiation of treatment. Compliance with evaluation of therapeutic response by physicians was measured at these same time intervals.

#### Acceptability.

Referring to implementers’ perception that the app is appealing, and it was considered either suitable.^19^^,^^31^ In this study, acceptability was measured qualitatively via semi-structured individual and group interviews at three levels: CHLs, health workers, and patients. Because of the Covid-19 pandemic, these activities were performed through videoconference or in person. Acceptability was evaluated based on three analytical categories: 1) relevance: whether the app is necessary, important, or useful for health needs, patients, and users; 2) suitability: whether the app is appropriate in the socio-health context; and 3) added value: whether the app has advantages or favorable differences with respect to routine or standard care.^31^^–^^34^

#### Usability.

This term refers to the degree to which the app can be used by CHLs and health workers to achieve the quantified objectives of effectiveness, efficiency, and satisfaction.^35^ In the current study, usability was evaluated using the same qualitative approaches as acceptability. Analytical categories that oriented the interviews were 1) attractiveness: the appealing presentation of the app to the users; 2) ease of use: ability of users to accomplish follow-up monitoring with the app; 3) speed of recording information: the time needed for users to record the data; 4) memorability: the ability of a user to use the app effectively on subsequent occasions; and 5) frequency of errors: how well the user can complete the respective task according to the user’s perception.^35^^–^^41^

## DATA ANALYSES

The management of data from these mHealth apps was described previously.^42^ Access was controlled by creating password-protected user accounts with encrypted passwords. Data transfers were also encrypted. Data collected by CHLs using the app and from interviews (particularly recordings and transcripts) were identified with a code to guarantee confidentiality. Clinical monitoring was performed three times during the study.

Quantitative analysis was performed using Stata 12 and R version 4.1.0 software. For categorical variables, groups were compared using either the χ^2^ or Fisher’s exact test, depending on the magnitude of the expected values. For dichotomous variables, odds ratios were calculated, with confidence intervals using Fay’s method,^43^ implemented in the “exact2 × 2” R package. Post hoc analysis was performed to evaluate possible confounding and effect modification by age, ethnicity, and type of medication. Crude and adjusted odds ratios were estimated between the effectiveness outcome (whether patient follow-up was done at 90 and/or 180 days) and study group (Guaral+ST app by CHL or standard of care). Confounding was determined to exist if the percentage change between the crude and adjusted ORs was 10% or greater.^44^ Stratum-specific odds ratios were calculated, and the Breslow-Day test was performed to evaluate homogeneity between strata.

For qualitative analysis, N-vivo software was used for coding. Following grounded theory procedures, the data were analyzed through a process of open, axial, and selective coding, identifying recurrent emerging themes, which were subsequently grouped into categories of analysis.^45^ The constant comparative analysis method was used to establish the differences and similarities between level of evaluations (CHLs, health workers, and patients)^46^ and study sites to ensure credibility. The coding process was carried out by a sociologist research assistant and was reviewed by the researcher who led the fieldwork to ensure confirmability.^47^

## RESULTS

### Enrollment and participant characteristics.

#### Characteristics of patients recruited in the quantitative analysis.

**Figure 3.**
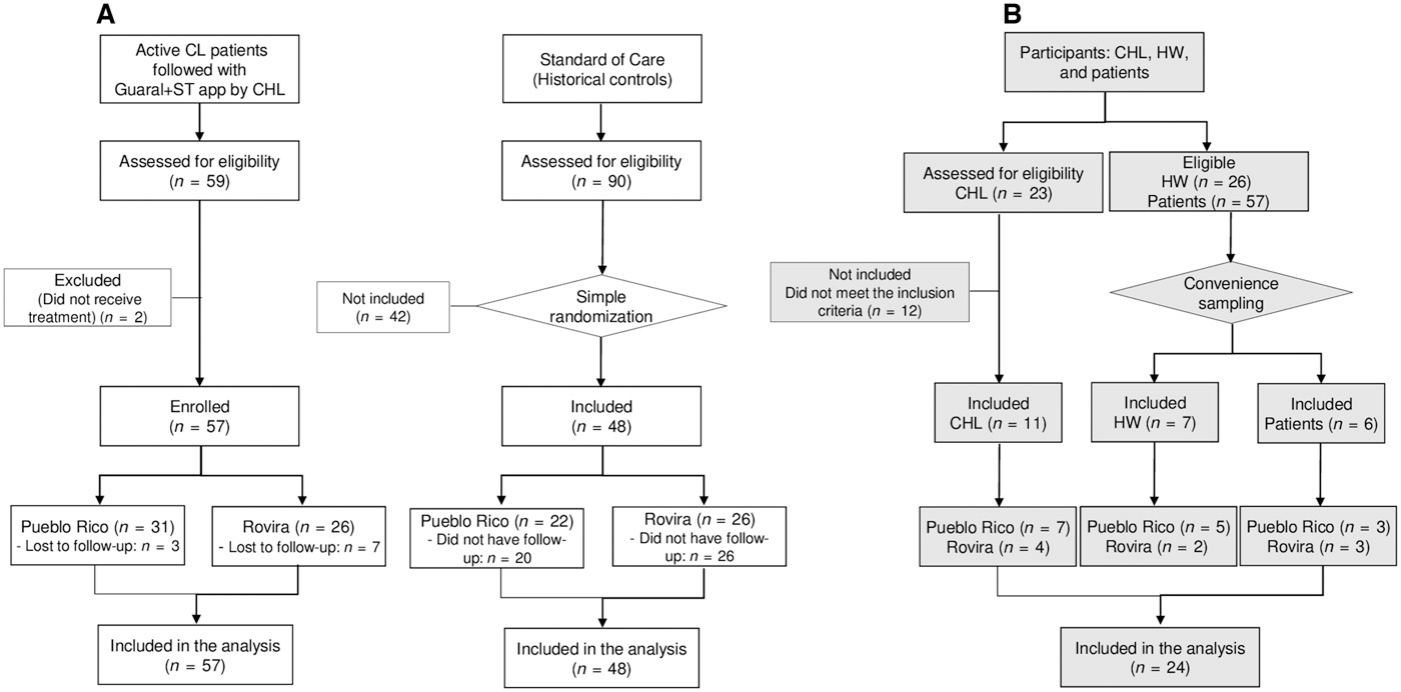
Schematic summary of the enrollment of study participants in quantitative and qualitative evaluations. (**A**) Evaluation of effectiveness: CL patients followed either by community health leader (CHL) using the Guaral: seguimiento a tratamiento (Guaral+ST) app or according to standard of care. (**B**) Evaluation of acceptability and usability at three levels: CHLs, health workers (HWs), and patients. CL = cutaneous leishmaniasis.

Participants were enrolled for quantitative analysis (effectiveness evaluation) at the two study sites; 57 patients with parasitologically confirmed active CL were prospectively followed up with the Guaral+ST app by CHLs, and 48 historical control patients were managed under standard of care–based follow-up. In the intervention group, 17.5% of patients (10/57) with CL were lost to follow-up before completing treatment (*n* = 1) or had incomplete follow-up (*n* = 9), whereas 96% (46/48) in the standard-of-care group had no follow-up after treatment (Figure 3).

Regarding the patients’ demographic and clinical characteristics, the distribution by gender was similar, with males proportionately higher in both groups, whereas differences in age, ethnicity, and treatment received were identified (Table 2). These differences reflect and underscore the difference in access of indigenous populations and young children in the study sites to health facilities. Pentamidine, which is administered in four doses, was prescribed to patients in Pueblo Rico with limited capacity to return to health facilities (pediatric and indigenous patients) for daily treatment during the 3 to 4 weeks required for treatment with MA or miltefosine, respectively.

**Table 2:** Demographic and clinical characteristics of patients with cutaneous leishmaniasis

Characteristics	Community-based strategy using Guaral+ST app (*n* = 57)	Standard of care (*n* = 48)	*P* value
Demographic characteristics			
Gender, *n* (%)			
Male	31 (54.4)	29 (60.4)	0.56*
Female	26 (45.6)	19 (39.6)	
Age (years), *n* (%)			
0–12	30 (52.6)	16 (33.3)	**0.05** *
> 12	27 (47.4)	32 (66.7)	
Ethnicity, *n* (%)			
Mestizo	31 (54.4)	36 (75.0)	**0.04** †
Indigenous	25 (43.9)	12 (25.0)	
Afro-Colombian	1 (1.7)	0 (0)	
Clinical characteristics			
Number of lesions, *n* (%)			
1–3	56 (98.2)	ND	–
> 3	1 (1.8)	ND	
Duration of oldest lesion (months), *n* (%)			
0–3	28 (49.1)	13 (27.1)	0.07*
3.1–5.9	7 (12.3)	9 (18.8)	
> 6	22 (38.6)	26 (54.1)	
Location of lesions, *n* (%)			
Head	22 (24.4)	ND	–
Trunk	7 (7.8)	ND	
Arms	30 (33.3)	ND	
Legs	31 (34.4)	ND	
Total lesions	90	–	–
Treatment, *n*(%)			
Meglumine antimoniate	25 (43.9)	45 (93.8)	**< 0.001** †
Miltefosine	2 (3.5)	2 (4.2)	
Pentamidine	30 (52.6)	1 (2.0)	

ND = No data available. *P* values in boldface mean that are statistically significant.

* χ^2^ test.

† Fisher’s exact test.

In terms of study site characteristics, the gender and age distributions in Pueblo Rico were similar for the community-based strategy using the app and the historical group attended at the local hospital. Differences were found between ethnicity and the treatment received. The predominant ethnicity in the intervention group was indigenous (81%; 25/31) followed by mestizo (16.1%; 5/31), whereas the historical control group was composed of 54.5% (12/22) and 45.5% (10/22) (*P* = 0.03), respectively. Regarding treatment, all patients in the community-based strategy group in Pueblo Rico received pentamidine, whereas 95.5% received MA in the historical patient control group (*P* ≤ 0.001). In contrast, no significant differences in demographic and clinical characteristics were found between the intervention and control study groups in Rovira. In Pueblo Rico, most participants in the community-based strategy group were children (74%; 0 to 12 years of age), whereas the opposite occurred in Rovira, where 73% of CL participants were > 12 years of age.

#### Characteristics of participants in the qualitative analysis.

Twenty-four participants were included in the qualitative analysis (acceptability and usability evaluation) (Figure 3). In Pueblo Rico, of the seven CHLs who participated, their ethnicity was mainly indigenous (5/7); only one was affiliated with the local hospital and another with the Departmental Secretary of Health. All health workers were mestizo and had completed technical education. Patients had received less formal education compared with health workers. In Rovira, all participants, six health workers, and three patients, were mestizo. The CHLs were affiliated with the local hospital and had a complete technical education as auxiliary nurses. Demographic characteristics of the CHLs, health workers, and patients who participated in the qualitative evaluation are detailed in Table 3.

**Table 3:** Demographic characteristics of participants in the evaluation of acceptability and usability

Study site	Participants	Ethnicity	Gender	Level of education
Pueblo Rico	CHLs (*n* = 7)	Afro-Colombian (*n* = 1) Indigenous (*n* = 5) Mestizo (*n* = 1)	Male (*n* = 6) Female (*n* = 1)	Incomplete university (*n* = 1) Completed technical education (*n* = 2) Incomplete technical education (*n* = 1) Completed secondary education (*n* = 3)
	Health workers (*n* = 5)	Mestizo (*n* = 5)	Male (*n* = 1) Female (*n* = 4)	Completed technical education (*n* = 5)
	Patients (*n* = 3)	Mestizo (*n* = 2) Indigenous (*n* = 1)	Male (*n* = 1) Female (*n* = 2)	Incomplete secondary education (*n* = 2) Completed primary education (*n* = 1)
Rovira	CHLs (*n* = 4)	Mestizo (*n* = 9)	Male (*n* = 1) Female (*n* = 3)	Completed technical education (*n* = 4)
	Health workers (*n* = 2)		Male (*n* = 1) Female (*n* = 1)	Completed university degree (*n* = 1) Completed technical education (*n* = 1)
	Patients (*n* = 3)		Male (*n* = 2) Female (*n* = 1)	No data (*n* = 2) No formal education (*n* = 1)

CHL = community health leader.

### Effectiveness of community-based strategy using the Guaral+ST app.

The proportion of patient who fulfilled follow-up at day 90 and/or 180 increased from 4.2% among historical controls (standard of care) to 82.5% among participants in the community-based strategy with the Guaral+ST app. The recording of treatment adherence, adverse drug reactions, and therapeutic response also increased significantly (*P* < 0.001). Adherence to treatment by > 90% was higher in the app group at 78.9% (45/57) compared with standard of care at 43.8% (21/48). Therapeutic response was not found in case histories of most of the patients (95.8%) whose follow-up was conducted under the standard of care in the historical control group. Twelve patients (12/57; 25.5%) in the app group failed treatment. Sixty-six percent (8/12) were children 0 to 12 years of age (Table 4).

**Table 4:** Effectiveness of community-based strategy using the Guaral+ST app

Effectiveness evaluation	Community-based strategy Guaral+ST app (*n* = 57)	Standard of care (*n* = 48)	Odds Ratio (95% CI)*	*P* value
Register of follow-up				
End of treatment	42 (73.7)	0 (0.0)	0 (0.000–0.030)	**< 0.001** †
Day 90 after initiating treatment	40 (70.2)	1 (2.1)	0.009 (0.000–0.065)	**< 0.001** †
Day 180 after initiating treatment	43 (75.4)	1 (2.1)	0.007 (0.000–0.051)	**< 0.001** †
Day 90 or 180 after initiating treatment	47 (82.5)	2 (4.2)	0.009 (0.001–0.047)	**< 0.001** †
Day 90 and 180 after initiating treatment	36 (63.2)	0 (0.0)	0 (0.000–0.048)	**< 0.001** †
Patients with adverse drug reactions registered				
Yes	22 (38.6)	4 (8.3)	0.144 (0.033–0.488)	**< 0.001** †
No	35 (61.4)	44 (91.7)		
Number of follow-up visits AST				
0	0 (0.0)	46 (95.8)	–	**< 0.001** ‡
1	7 (12.3)	2 (4.2)	–	
2	12 (21.0)	0 (0.0)	–	
> 3	38 (66.7)	0 (0.0)		
Registration of treatment adherence				
Yes	56 (98.2)	26 (54.2)	0.021 (0.001–0.149)	**< 0.001** ‡
No	1 (1.8)	22 (45.8)		
Treatment adherence				
< 90%	11 (19.3)	5 (10.4)	–	**< 0.001** †
> 90%	45 (78.9)	21 (43.8)	–	
Unknown	1 (1.8)	22 (45.8)	–	
Registration of therapeutic response at day 90 or 180 AST				
Yes	47 (82.5)	2 (4.2)	0.009 (0.001–0.048)	**< 0.001** ‡
No	10 (17.5)	46 (95.8)		
Therapeutic response at day 90 or 180 AST (*n* = 49)				
Definitive cure	35 (74.5)	0 (0.0)	NC	0.077‡
Failure	12 (25.5)	2 (100)	–	

AST = after start of treatment; NC = not calculable; OR = odds ratio; Guaral+ST app = Guaral: seguimiento a tratamiento. *P* values in boldface mean that are statistically significant.

* For dichotomous variables, standard of care relative to app.

† χ^2^ test.

‡ Fisher’s exact test.

Treatment failures were concentrated in pediatric patients. Therapeutic failure with pentamidine was 29.6% (8/27). Of the eight failures, six were children 12 years of age or younger. Failure for MA was 16.7% (3/18). The only patient who failed treatment among the four receiving miltefosine was 3 years of age (Supplemental Table 1).

Data on adverse events (AEs) were available for more participants in the mobile application group than in the historical group: 22/57 (101 events) compared with 4/48 (50 events). Most AEs were mild. Among patients followed with the app, 17 received MA and 5 received pentamidine. Among these 22, the most frequent AEs for MA (*n* = 94) were injection site reactions, arthralgia, headache, and myalgia. Fifty-nine percent of these patients had 1 to 5 events, with the remaining 41% having 6 to 11. Among the seven AEs registered, in patients receiving pentamidine were dizziness and fever. In the historical group, the three patients who received MA reported 39 AEs (12 to 14 each). The remaining patient with AE data in this group received miltefosine and reported 11 cevents (including vomiting, nausea, dizziness, and headache).

Post hoc analysis showed confounding by age, ethnicity, or treatment medication between groups, with the percentage changes between crude and adjusted odds ratios being higher than 10% (Supplemental Table 2). After adjustment, however, associations remained between patient follow-up at day 90 or 180 and the group (app or historical).

### Implementation outcomes.

#### Fidelity.

**Figure 4.**
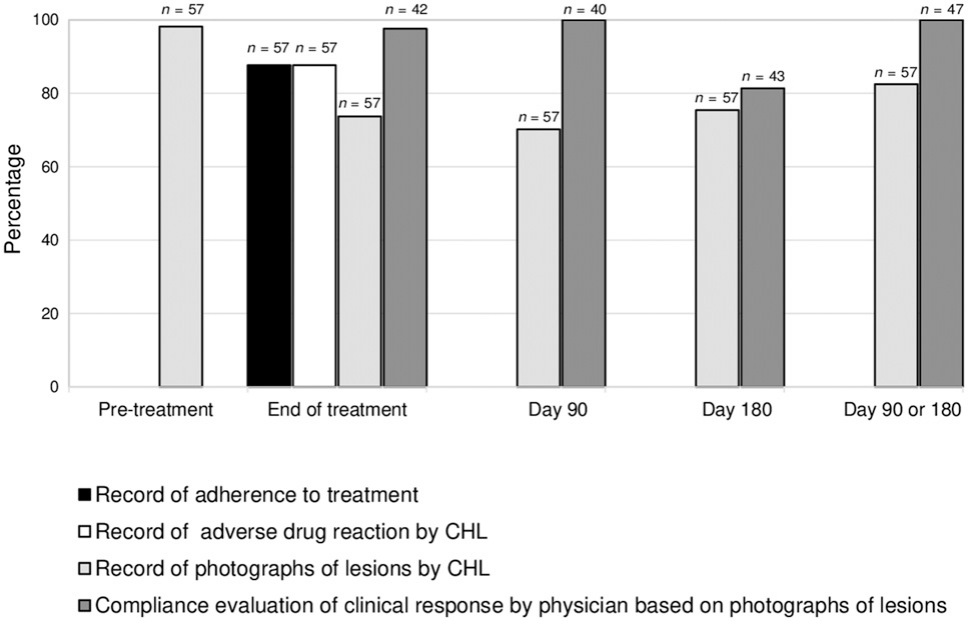
Fidelity of CHL and physician to the implementation strategy. CHL = community health leader.

Overall, fidelity to the strategy using the app was between 70% and 100% for the recording of 1) adherence to treatment (87.7%); 2) adverse reactions (87.7%); 3) photographs of lesions taken by CHL before treatment, at end of treatment, and at day 90 or 180 (98.2%, 73.7%, and 82.7%, respectively); and 4) evaluation of therapeutic response at day 90 or 180 by study physicians via photos taken by CHLs (100%) (Figure 4).

Both recordings of adherence by CHLs using the app based on medication doses received and adverse drug reactions were similarly fulfilled at 87.7%, as these variables were captured together within the same app follow-up session. According to the protocol, patients should have been followed up weekly by CHLs until the end of treatment. Some patients who lived in particularly remote rural areas were, in fact, followed up at 2-week intervals or at an unprogrammed time according to their availability. Adherence of CHLs in obtaining lesion photos and evaluation of clinical response by the study physician at days 90 and 180 ± 2 weeks after the start of treatment was 82.5% and 100%, respectively (Figure 4).

#### Evaluation of the acceptability of the app by community health workers, health workers, and patients.

The app was considered acceptable by all participants. The principal themes that emerged were CL management, health care in rural areas, efficiency of health institutions, CL monitoring, technological innovation, and opportunities to use the app for other diseases. Participants emphasized the importance of facilitating patient follow-up, case detection, expediting health workers’ tasks, and data collection (Supplemental Table 3).

With respect to the analytical category, relevance, CHLs and health workers stated that the app facilitated CL management. As the app’s main benefit, they highlighted how follow-up was more efficient and easier to achieve because they were able to record changes in the lesions via photographs and monitor any adverse drug reactions. All participants in the qualitative analysis stated that the app allowed for adequate patient care in rural areas, as it eliminated the need for patients to make long and costly trips to the medical center. As an additional benefit, CHLs and health workers pointed out the possibility of detecting additional cases of CL because visiting communities for patient follow-up allowed contact with the population and identification of new cases:

“As a health worker […] one goes back to the patient’s home and the follow-up is not only for them but also their family and the people around them or within their community who may also have leishmaniasis, so the app also helps detect cases.” (Group interview. CHL/Health worker. December 4, 2020).

In relation to suitability, the participants stated that the app improved the efficiency of health care providers. Community health leaders and health workers pointed out that the app improved their work in the rural context by incorporating new approaches and technology into their efforts. During home visits, the app also provided them with consolidated information for each patient and did not require the use of paper, which can be cumbersome and vulnerable during fieldwork.

“[…] all the information is saved there [in the app] and there’s no risk that, on the way to visit the patients, paper documents get damaged and we lose information” (Focus group 01. CHL. April 26, 2021*).*

For health workers, the app improved the relationship between patients and institutions because of how positively the community values institutional interest in their health; treatment monitoring through regular follow-up visits improved community perception of institutional support and care. Following up patients with the app increased patients’ motivation, facilitated the health workers’ tasks, and improved patients’ health conditions:

“They feel support since it’s more supervised because before they were evaluated to see whether it (the lesion) had healed or not after a while, whereas now it is almost every day, there is more commitment to the patient, and for them they feel that support […] ” (Focus group 01. CHL. April 26, 2021).

In terms of the added value analytical category, CHLs and health workers in both municipalities said that the app improved the monitoring of disease progression and treatment by generating photographic evidence and collecting data on adherence, thereby providing a detailed record of the therapeutic response and AEs and an overall understanding of the entire treatment process for each patient:

“With the app we realize if the person is healing and how long it’s taking to heal, and if it is failing, the new lesions, […] those people for whom the treatment fails and who otherwise would not be detected” (Group interview 05. CHL/Health worker. December 4, 2020).

Community health leaders and patients highlighted the usefulness of the photographic evidence of disease evolution during treatment and the value of the opportune detection and information collected on adverse reactions. Health workers, for their part, valued the app as a technological innovation and its practicality for the health sector in the rural context:

“…with the app the follow-up is very good, especially because of the photos, and because the information is kept there, it is very continuous, then above all, the evidence of the photographic record is very important, if one did it manually, that is, a lot is lost…” (Interview 05. CHL/Health worker. August 19, 2020).

In terms of other uses of the app, the CHLs in both municipalities noted that it would be pertinent to apply this mHealth strategy to management of other diseases. Health workers in Pueblo Rico expressed interest in establishing a departmental network in which the photographic record would be sent from rural communities and assessed by physicians at the first-level (primary care) administrative division (Department). In addition, they pointed out the app’s potential use by health workers affiliated with health institutions.

#### Evaluation of usability of the app by CHLs and health workers.

Overall, CHLs and health workers expressed a positive perception of the app’s usability. Participants considered that the follow-up app was easy to use and to remember and that the process of recording information was rapid. They also mentioned limitations, especially in Pueblo Rico, due to Internet connection coverage and the CHL’s ability to use a cell phone and the app (Supplemental Table 4).

In relation to the attractiveness analytical category, CHLs and health workers in both municipalities mentioned that they liked the design of the app and stated that it did not need changes. Community health leaders, who were the main users of the app, noted that the app has attractive colors, a legible font, and good graphic representations, which facilitate its use.

Regarding ease of use, CHLs in Pueblo Rico reported that it was easy to monitor patients and record adverse effects. Community health leaders in Rovira mentioned that data input, taking photos, and localization of cutaneous lesions were the easiest steps:

“The easiest part is recording the administration of treatment, it [the app] says the day and how many vials, it’s really easy. Also, registering the daily symptoms that the patient may have, like fever, headache.” (Focus Group 02. Community health leader. November 27, 2020).

In terms of the memorability, all participants in both municipalities found that with practice, the procedure was easy to recall and agreed that the app’s step-by-step directions facilitate recall:

… “The steps are there. We know that if we ask the series of questions, we have to wait to start taking the photos, then you know more or less what you have to do. With the questions, every question leads you”. (Interview 01. Community leader. August 19, 2020).

With regard to the speed of recording information, for CHLs in Pueblo Rico, there were aspects that some considered fast and others slow, suggesting that they may have been isolated incidents or associated with each leader’s proficiency in the use of cell phones and the app. However, there was a clear trend in the perception of the rapidity of data registration, especially the questions about patient follow-up and adverse effects.

In contrast, among CHLs in Pueblo Rico, synchronization (i.e., uploading of data from the phones to the server where it can be accessed remotely) was a difficult feature. This is a common problem in all analytical categories of usability, as synchronization was identified by app users as frequently generating delays. Specific processes linked with connectivity include uploading photos, patient assignment, and app log-in. Issues with synchronization were related to limitations in Internet connection in both Pueblo Rico and Rovira according to a CHL:

“The only hard thing is the synchronization… to synchronize, myself, I find it complicated, because you always have to have data [in your cellphone plan] or be connected to the Wi-Fi network, and synchronizing consumes a lot of data, and connecting to the network is slow… or where I am, it takes quite a while to synchronize” (Interview 06. Community leader. August 19, 2020).

In terms of frequency of errors, CHLs in both municipalities mentioned that the errors were initially due to user mistakes but were later overcome. As for health workers, they noted that if they paid attention when using the app, there were no errors and that in addition, the app provides the option to correct the user errors before saving data.

## DISCUSSION

Our findings indicate that this community-based strategy using the Guaral+ST app improved the follow-up of CL patients in rural settings in Colombia. The proportion of patients with follow-up visits increased from 4.2% under standard of care to 82.5% using the app (*P* < 0.001). The effect of the community-based strategy using the app also expanded information on treatment adherence, adverse drug reactions, and therapeutic response (*P* < 0.05 for all comparisons). Regarding implementation outcomes, in this mixed-methods study, we determined from the CHL, health worker, and patient perspectives that the app was accepted because it met a public health need and was suitable for use in rural areas. It was perceived as easy to use, particularly regarding data input, and photographic recording of lesions was considered easy to perform. Overall, the app and its use by community leaders and health workers facilitated case management, patient follow-up, and access to clinical results, including treatment effectiveness.

One of the knowledge gaps in real-life CL management and care is the lack of data on treatment effectiveness. In the Americas, 38.5% of cases lack information on treatment outcomes, and in Colombia and several other countries of the region, this information is unavailable.^4^ The Guaral+ST app demonstrated potential in reducing this gap, particularly considering that Colombia continues to improve connectivity, particularly in urban centers, and has committed to bringing Internet connectivity to the entire country by 2025.^48^ The findings substantiate the feasibility and contribution of remote evaluation of treatment to overcoming the geographic and economic barriers to medical care that CL patients often face.^7^^,^^49^ Guaral+ST and other mobile applications for NTDs improve access to medical assistance and monitoring of clinical results at the community level.^9^ These results align with the WHO NTD roadmap.^2^

The Guaral+ST app, which was perceived as relevant, suitable, and adding value to monitoring CL patients, had high acceptability. These perceptions are facilitators for wider adoption and potential scale-up of the app.^50^ The use of human-centered design principles in the development of this tool likely facilitated its ease of use, as well as the application’s fitness within the local population and context.^15^ Other facilitators described for different mHealth apps used in diverse interventions include user-centered design, motivational aspects for the user, effective marketing, humanizing technology, accessibility, and patient–provider interaction oriented to personnel values.^51^^–^^53^ Another factor that will facilitate the adoption of this intervention is its alignment with the Colombian treatment guidelines for CL.^6^

Application usability was positively valued: The step-by-step workflow and graphic instructions and illustrations facilitated its use. However, usability was negatively affected by limited connectivity, which impacted data synchronization. Limited access to the Internet in rural areas is a potential barrier to mHealth. Variance in Internet service between rural and urban areas is wide in Colombia; at the end of 2020, 56.5% of the population had Internet access in large cities compared with only 23.8% in rural and remote rural areas.^54^ Nevertheless, this limitation was managed through the offline capture of data by the app and data synchronization when the user was in a location with Internet service. Notably, the Guaral+ST app was evaluated and perceived as easy to use by CHLs with relatively limited formal education (mostly nonprofessionals), supporting its potential for wider use in community-based and primary levels of care.

We observed that participants in Pueblo Rico had more difficulties in using the technology than those from Rovira and needed more training. Overall, barriers to usability included institutional capacity of the local health system, lower network coverage, differences in education levels, technological literacy, and Spanish language proficiency (especially among indigenous populations), as described elsewhere.^52^^,^^55^^,^^56^ Implementation and scaling of skin NTD mHealth apps should take into account local needs and context, perceived utility and simplicity, users’ training needs and empowerment, technological requirements to ensure a long-term mHealth platform, cost assessment, and a monitoring and evaluation process.^3^^,^^57^

Fidelity to the strategy of the different users (CHLs, health workers) was between 70% and 100% for recording of lesion photographs, adherence to treatment, adverse drug reactions, and evaluation of clinical response by physicians based on photos. These results support the viability of implementation of this strategy for monitoring patients with CL in remote rural areas. Although the strategy called for weekly monitoring of adherence to treatment and adverse drug reactions during treatment, in some cases, the follow-up was performed at a different interval according to circumstances. The community-based approach involving community members as CHLs offers flexibility in the programming of follow-up.

A particular strength of this study was the use of mixed methods to estimate the intervention’s effectiveness and to subsequently understand participants’ attitudes toward use of the app and difficulties with the technology. This information is critical to the scale-up of the intervention. The design also facilitated the interdisciplinary integration of health and social sciences.

The main weakness in the evaluation of effectiveness was the nonrandom allocation for the intervention group using the app. Historical patients were included as a control group considering the difficulties, time, and cost of implementing the randomization process in the field, as well as ethical concerns. Participants in the historical control group were selected by a simple randomization process to minimize selection bias. Differences were found in baseline characteristics between the two groups (community-based strategy with the app and standard of care) in terms of age, ethnicity, and treatment. Post hoc analysis showed positive confounding in relation with the first two variables, age and ethnicity, and negative confounding in the treatment. Differences between the two groups also suggest that the population that consults and reaches the health system through the passively administered standard of care is different from the patient population accessed by active community-based monitoring. Most of the patients in Pueblo Rico were children and of indigenous ethnicity, which facilitated the provision of health services to these vulnerable populations.

Qualitative evaluation and data collection were performed virtually. Connectivity issues, communication barriers, and cultural characteristics of Pueblo Rico represented challenges for virtual data collection and may have impacted the study participants’ responses. The use of virtual tools for qualitative data collection has been widely implemented and described by social scientists in their research.^58^^,^^59^ During the Covid-19 pandemic, recommendations for collecting information virtually, ways to enhance platform security, and resources for the virtual collection of data were developed to adapt to social distancing and mobility restrictions.^60^

Interactions with local authorities and stakeholders during and after this study encouraged implementation of mHealth strategies to improve health system access to dispersed communities and vice versa. The cost of the intervention, its sustainability, and the interoperability between the app and the information systems of local hospitals were not assessed within the scope of the current study, yet receptivity has been engendered. These issues, as well as organizational and programmatic procedures in place, are important considerations in the scale-up and sustainability of mHealth according to the WHO model.^61^ They will be the focus of the next phase of the implementation of mHealth interventions.

Mobile technology offers opportunities for improving services provided by community health workers to manage diverse illnesses (maternal and child health, HIV/AIDS, and sexual and reproductive health). The most common mobile apps are focused on ensuring community health workers’ compliance with standards for health services, emergency referrals, receiving alerts and reminders by community health workers and caregivers, collecting patient data through mobile phones, and community education and training.^10^^,^^62^^,^^63^

Several apps targeting health care professionals and patients have been developed for clinical management and epidemiological surveillance of NTDs in low- and middle-income countries settings.^3^ Among these is the skin NTD app developed by the WHO to facilitate the diagnosis and clinical management of patients with diverse transmissible dermatological conditions. This app is aimed principally at health care workers.^12^ Similarly, the LeishCare app was developed to allow health care professionals to record the evolution of CL skin lesions using photos, calculate a score that is defined to identify the risk of death from visceral leishmaniasis, and provide guidance in the management of these diseases in settings where both occur.^11^ SkinApp supports health workers in peripheral locations in recognizing the early signs and symptoms of skin diseases, including skin NTDs, and to opportunely initiate treatment or refer patients for more advanced diagnostic evaluation.^13^ The Guaral+ST app evaluated in this report aims to assist and empower CHLs and health care professionals in monitoring treatment of patients in remote rural areas. Future efforts will focus on scale-up and evaluation of penetration and sustainability. The future development of an app for direct interaction of patients with health providers, including transmission of photographs of lesions to health workers for clinical evaluation by medical personnel, could also build upon the findings of this investigation.

The intervention with the app allows clinical monitoring of CL patients. The effectiveness of MA was similar to that reported in other publications.^64^^–^^66^ Among the eight patients who received pentamidine and failed treatment, it is noteworthy that 6/8 patients were children. Clinical trials to evaluate pentamidine’s efficacy in the pediatric population are needed.

This study was a collaborative effort between the research team, affected communities, and health system authorities sponsored by a pharmaceutical company. Future global health innovation for NTDs will require intersectoral collaboration among investigators and other stakeholders to respond to patient needs. Implementation research and innovative, integrated approaches to diagnose and treat leishmaniasis and other NTDs using mHealth technologies developed, evaluated, and implemented in partnership with communities, health authorities, and multidisciplinary research teams can bridge the gap in access to diagnosis and treatment.

The Guaral+ST app is an effective, easy-to-use mHealth tool that is acceptable for community leaders and health volunteers for monitoring and determining the effectiveness of treatment of CL. The community-based approach used in this study alleviates geographic barriers to the monitoring of interventions in areas where populations are dispersed and medical personnel are scarce. Use of the mHealth app by CHLs provided a link between health personnel in health facilities and patients in their rural communities for the exchange of actionable information.

## Supplemental Materials


Supplemental materials
