# Supplementary material for: mHealth Monitoring of Treatment of Cutaneous Leishmaniasis Patients: A Community-Based Implementation Study
Source: Am J Trop Med Hyg. 2023 Aug 28;109(4):778–90. doi: 10.4269/ajtmh.22-0805 (PMC10551068; doi:10.4269/ajtmh.22-0805)
Supplement: Supplementary file 1 [file tpmd220805.SD1.pdf]

S1 Table. Therapeutic response determined using photographic monitoring by CHL with the application and evaluated by the study physician

Age	0 - 12	> 12	Total
Pentamidine, n (%)			
Cure	16 (84.2)	3 (15.8)	19
Failure	6 (75.0)	2 (25.0)	8
Total	22	5	27
Meglumine antimoniate, n (%)			
Cure	4 (26.7)	11 (73.3)	15
Failure	1 (33.3)	2 (66.7)	3
Total	5	13	18
Total	27	18	45

Two patients who received miltefosine were not included in the table. One of them failed treatment. Dosing scheme for pentamidine: 4mg/kg intramuscular, administered in 4 doses

S2 Table. Evaluation of confounding and effect modification between follow-up at day 90 or 180 after initiation treatment, age, ethnicity and treatment

	Odds ratios for comparisons between study groups						
	Crude OR		Adjusted OR		Mantel-Haenszel stratified OR		
**Variables**	**OR**	**(95% CI)**	**OR**	**(95% CI)**	**OR**	**(95% CI)**	**p***
**Age (years)**							
0 - 12	0.009	(0.001 - 0.048)	0.002	(0.000 - 0.039)	0.005	(0.000 - 0.072)	0.479
>12					0.000	(0.000 - 0.066)	
**Ethnicity**							
Afro-Colombian	0.009	(0.001 - 0.048)	0.003	(0.000 - 0.043)	NC	NC	0.624
Indigenous					0.008	(0.000 - 0.128)	
Mestizo					0.000	(0.000 - 0.048)	
**Treatment**							
Meglumine antimoniate	0.009	(0.001 - 0.048)	0.015	(0.003 - 0.078)	0.018	(0.002 - 0.108)	0.715
Miltefosine					0.000	(0.000 - 0.922)	
Pentamidine					0.000	(0.000 - ND)	

*Breslow-Day test for homogeneity of OR

NC: not calculable, only one person had ethnicity Afro-Colombian in the Guaral+ST app group

ND: Not defined

S3 Table. Acceptability evaluation: Analytical categories and emerging topics by qualitative analysis

Analytical Categories	Principal themes	Emerging Themes		
		CHL	HW	Patients
Relevance	CL management	▪ **Follow up**<br>▪ Case detection	▪ **Follow up**<br>▪ Case detection	▪ **Follow up**
	Health Care in rural areas	▪ Displacement	▪ Medical assessment	▪ Displacement
Suitability	Efficiency of health institutions	▪ Health worker´s job<br>▪ Patient-institution relationship	▪ Health worker´s job	▪ Patient´s health
Added value	CL monitoring	▪ Treatment behavior<br>▪ Data collection<br>▪ Adverse effects<br>▪ Lesion evolution<br>▪ Adherence	▪ Treatment behavior<br>▪ Data collection<br>▪ Therapeutic response<br>▪ Lesion evolution	▪ Therapeutic response<br>▪ Injury evolution<br>▪ Adherence
	Technological innovation	▪ Photographic record	▪ Innovation<br>▪ Therapeutic response	▪ Photographic record<br>▪ no difference
	Other uses of the app	▪ Other diseases<br>▪ Other health procedures	▪ Departmental network<br>▪ App users<br>▪ Other health procedures	

The **bold font** implies a high concentration of data**,** the underline implies a medium concentration of data, and the regular font implies a low concentration of data.

S4 Table. Usability evaluation: Analytical categories and emerging topics by qualitative analysis

Analytical Categories	Emerging themes	
	**CHL**	**HW**
**Attractiveness**	▪ <u>Good design</u><br>▪ Nice colors<br>▪ Useful graphic representations<br>▪ Suitable layout	▪ <u>Good design</u><br>▪ Nice colors
**Ease of use**	▪ <u>Follow up tools</u><br>▪ <u>Ease of photo-taking</u>	▪ **Follow up tools**<br>▪ <u>Data registration easy</u><br>▪ Step by step tools<br>▪ Difficulties of photo-taking
**Speed of Registration**	▪ <u>Data entry is fast</u><br>▪ <u>Fast Patient follow up</u><br>▪ Patient assignment delays<br>▪ **Synchronization delays**<br>▪ Photo upload delays<br>▪ **App login delays**	▪ **Very fast**
**Memorability**	▪ <u>Practice needed</u><br>▪ <u>Step by step tools</u><br>▪ <u>Coordinator's help</u>	▪ **Practice needed**
**Frequency of errors**	▪ Errors opening the app<br>▪ No errors	▪ Pay attention<br>▪ Fixable mistakes<br>▪ No errors

The **bold font** implies a high concentration of data**,** the <u>underline</u> implies a medium concentration of data, and the regular font implies a low concentration of data
